# FOXP1-GINS1 axis promotes DLBCL proliferation and directs doxorubicin resistance

**DOI:** 10.7150/jca.85906

**Published:** 2023-07-16

**Authors:** Zhenfa Chen, Ting Wang, Cui Li, Wei Zhang, Wenbin Huang, Jun Xue, Jundong Wang, Shufeng Li

**Affiliations:** 1 Key Laboratory of Developmental Genes and Human Disease in Ministry of Education, Jiangsu Provincial Key Laboratory of Critical Care Medicine, Department of Biochemistry and Molecular Biology, Medical School of Southeast University, Nanjing 210009, China.; 2 Department of Pathology, Nanjing First Hospital, Nanjing Medical University, Nanjing 210006, China.; 3 Department of Hematology, Nanjing First Hospital, Nanjing Medical University, Nanjing 210006, China.; 4 Department of ultrasound, Nanjing First Hospital, Nanjing Medical University, Nanjing 210006, China.

**Keywords:** FOXP1, GINS1, DLBCL, transcription, B cell

## Abstract

GINS1 is overexpressed in several types of cancers including leukemia and linked to poor outcomes. However, GINS1 remains poorly investigated in DLBCL (diffuse large B-cell lymphoma). This project aimed to explore the expression, functions and regulation of GINS1 in DLBCL. In this study, through analysis of clinical specimens from DLBCL patients, we uncovered that GINS1 was upregulated in DLBCL. By EMSA, ChIP and luciferase reporter assays, it was found that FOXP1 transcriptionally activated GINS1 expression by directly binding to the promoter region of the GINS1 gene. Western blotting and RT-PCR also revealed that GINS1 expression positively correlated with FOXP1 in human DLBCL specimens and cell lines. In an in vivo xenograft lymphoma mouse model, the FOXP1/GINS1 regulatory axis was also validated. Moreover, with CCK8 cell proliferation assays and colony formation assay, elevated GINS1 expression was found to be associated with doxorubicin resistance in lymphoma cells. Our findings showed that the FOXP1-GINS1 axis played a critical role in DLBCL development and doxorubicin resistance, and targeting the FOXP1-GINS1 axis could be a potential therapeutic approach for DLBCL treatment.

## Introduction

DLBCL is a kind of aggressive lymphoma that is composed of large, transformed B cells and displays a diffuse growth pattern. Diffuse large B-cell lymphoma (DLBCL) is the most common type of non-Hodgkin lymphoma worldwide 1. Despite the application of the current frontline chemotherapy regimen of rituximab, cyclophosphamide, doxorubicin, vincristine and prednisone (R-CHOP), which leads to cure in approximately 60% of patients, not all patients benefit from these treatment options. Up to 40% of DLBCL patients experience relapse or exhibit refractory disease, and chemoresistance remains the most urgent challenge in the clinical management of DLBCL patients. Thus, elucidating the mechanism underlying DLBCL chemoresistance and identifying new therapeutic targets are urgently needed to improve the treatment response.

GINS1, also known as PSF1, is a member of the heterotetrametric GINS complex. The GINS complex, which contains the four subunits GINS1, GINS2, GINS3 and GINS4, is essential for both the initiation and progression of DNA replication in eukaryotes^2^.

Dysregulation of the members in the GINS complex was revealed to correlate with the progression and prognosis in diverse cancers^3^. In particular, upregulation of GINS1 has been reported in leukemia, non-small cell lung cancer, and breast cancer^4^-^7^. Due to its robust clinical implications, GINS1 has recently received increasing attention.

FOXP1 is a transcription factor of the FOX gene family, named for the forkhead-box DNA-binding domain present in the gene family. Accumulating evidence reveals that FOXP1 has a broad range of functions. FOXP1 is widely expressed and has been shown to have a role in cardiac, lung and lymphocyte development^8^, ^9^. FOXP1 is targeted by recurrent chromosome translocations and FOXP1 overexpression represents a biomarker for poor prognosis in several types of lymphomas^10^, ^11^. A variety of B-cell tumors express abnormal levels of FOXP1^12^, ^13^. FOXP1 overexpression defines a group of lymphomas with a poor-prognosis, but the underlying molecular mechanism remains to be elucidated.

Here, in this study, we showed that GINS1 was regulated by FOXP1. GINS1 expression was critical for DLBCL cell proliferation and doxorubicin resistance. Downregulation of FOXP1/GINS1 significantly sensitized lymphoma cells to doxorubicin. Our study established the functional relevance between FOXP1-GINS1 and drug resistance in DLBCL. FOXP1-GINS1 could be a target for overcoming drug resistance in DLBCL.

## Material and Methods

### Cell lines

DLBCL cell lines (FARAGE, DB and SU-DHL-2) and normal lymphocytes (IM-9) as well as a human embryonic kidney cell line (HEK-293 T) were used for this study. FARAGE and SU-DHL-2 cells were exposed to incremental doses (0.0035, 0.035, and 0.35 μM) of DOX (Sigma-Aldrich, Merck KGaA, Darmstadt, Germany). The DOX-resistant cells FARAGE/DOX and SU-DHL-2/DOX were established when they could stably grow under 0.35 μM DOX treatment^14^.

### DLBCL patients

This project was reviewed and approved by the Ethics Committee of the Nanjing First Hospital. All the specimens included in this study were collected from patients who presented to the Nanjing First Hospital with newly diagnosed, previously untreated DLBCL from September 2020 to August 2022 (Supplementary Table S1). Informed consent forms were obtained from all the patients. Inclusion criteria included that all patients had complete clinical data. The diagnosis was confirmed by immunohistochemistry according to the 2016 WHO diagnostic criteria for DLBCL. None of the patients exhibited any additional tumors. Patients who had undergone radiotherapy or chemotherapy were excluded.

### RT-qPCR

RNA was isolated and cDNAs was synthesized. Real-time PCR was performed with SYBR Premix Ex TaqTM II. Relative expression levels of mRNA were calculated using the 2^-ΔΔCt^ method. Primer sequences are shown in Supplementary Table S2.

### Transfection, lentiviral particle formation and infection

GINS1 and FOXP1 expressing plasmids were constructed based on pCDH-CMV-MCS-EF1-copGFP-T2A-puro. pLKO.1-shFOXP1 or pLKO.1-shGINS1 vectors were constructed to express short hairpin RNA (shRNA) (Supplementary Table S3). To produce the recombinant viruses, 293T cells were transfected with the above recombinant lentiviral vectors and packaging plasmids. Lentiviral particles were harvested 48 h after transfection. DLBCL cells were infected with the collected viral supernatant and incubated for 48 h. After 48 h, infected cells were selected with puromycin.

### Electroporation and luciferase reporter gene assay

Luciferase reporter plasmids with the luciferase gene under transcriptional control of the GINS1 promoter were constructed as described previously 15**.** SU-DHL-2 cells were transiently transfected by electroporation using the AMAXA® Cell Line Nucleofector® Kit V (Lonza, Cologne, Germany)^16^. Briefly, cells were resuspended in 100 µL of nucleofector solution V. Two micrograms of luciferase reporter plasmid DNA was added to each cell suspension and transferred to an Amaxa-certified cuvette. Electroporation was performed. The luciferase reporter assay was performed as described previously 15.

### Clonogenic methylcellulose assays

DLBCL cells (800 cells) were plated in 60-mm dishes with methylcellulose-based media purchased from R&D Systems Inc.^17^. After 15 days, the number of colonies (over 20 cells) in each dish was counted under a microscope.

### Expression and purification of recombinant FOXP1 protein

The plasmid pET28a-FOXP1 was transformed into E. coli BL21, and recombinant FOXP1 expression was induced by IPTG. Then, the cultures were centrifuged, and the cell pellet was collected and sonicated. Recombinant FOXP1 protein was purified through NI-NTA affinity chromatography.

### Electrophoretic mobility shift assay (EMSA)

Nuclear extracts from cells were obtained. Synthetic biotin-labeled oligonucleotides were synthesized, and oligonucleotide sequences are shown in supplementary Table S4. The probes of annealed oligonucleotides were subsequently incubated with nuclear extract or purified protein. The DNA-protein complex was separated from nondenaturing polyacrylamide, and detected with HRP-conjugated streptavidin.

### Chromatin immunoprecipitation (ChIP) assay

ChIP assays were performed as previously described^18^. Cells were fixed and resuspended in cell lysis buffer. Then, chromatin was sonicated and incubated with antibody, and protein A+G agarose beads were added. DNA fragments were purified for PCR analysis. The primers are shown in Table S4.

### Western blot

Tissue or cellular protein was extracted with RIPA lysis buffer. Western blotting was conducted with antibodies specific for FOXP1, GINS1, Tubulin, Ki67 and Flag (Table S5).

### CCK-8 assays

Cell Counting Kit-8 was used for the cell proliferation assay according to the manufacturer's instructions.

### Xenograft lymphoma model

Male BALB/c-nude mice (six weeks old) were purchased (Gempharmatech Company, China). Animal welfare and experimental procedures were performed in accordance with national and institutional guidelines, and all experiments were approved by the Animal Experimentation Ethics Committee of Southeast University. Each mouse was injected subcutaneously with 8×10^6^ cells. Tumor size was monitored. Finally, tumors were obtained and weighed.

## Results

### Expression of the GINS complex in DLBCL was upregulated

**Figure 1:**
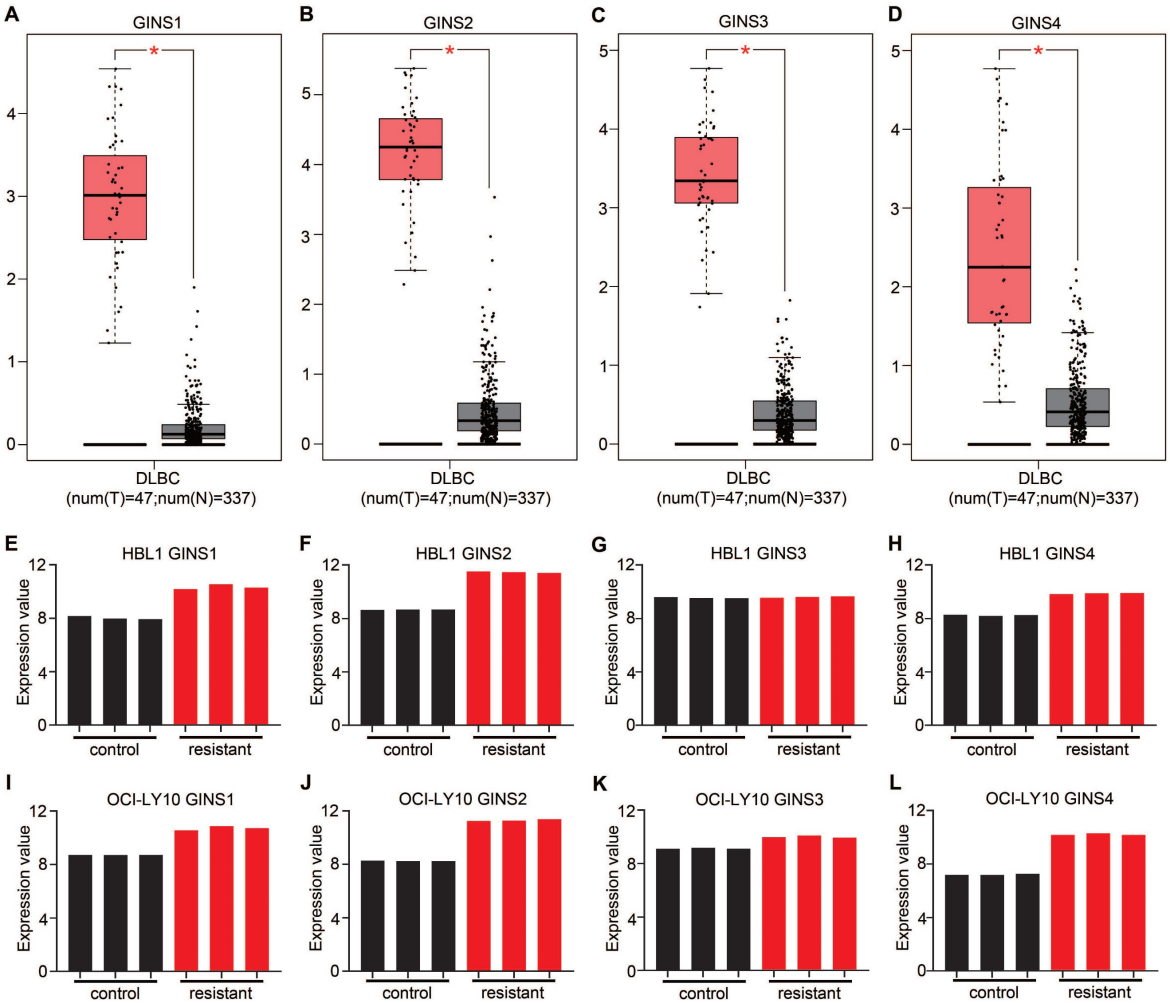
The mRNA expression of GINS complex and the correlation between GINS expression and drug resistance. Comparative expressions of **(A)** GINS1, **(B)** GINS2, **(C)** GINS3 and **(D)** GINS4 in DLBCL and normal control tissues by GEPIA. *p-value < 0.05.** E-H.** Expression of GINS complex in HBL1 parental cells and Ibrutinib resistant cells.** I-L.** Expression of GINS complex in parental OCI-Ly10 cells and Ibrutinib resistant cells.

The GINS complex has been implicated in the prognosis of various cancers. However, based on the current understanding, the functional relevance between the GINS complex and DLBCL drug resistance has not been investigated. The emergence of bioinformatics tools has provided convenient, credible, methods to analyze expression profiles. Analysis of data in the GEPIA database revealed an overexpression of mRNA for each member of the GINS complex in DLBCL tissues compared to normal control tissues (p-value <0.05) (Figure 1A-D). More interestingly, the GSE138126 dataset was analyzed, which contains expression profiles for parental and ibrutinib-resistant DLBCL cell line clones 19. The differential expression analysis showed that almost every member of the GINS complex was upregulated in resistant cells compared to parental HBL1 and OCI-Ly10 cells (Fig. 1E-L). These results indicated that the expression level of the GINS complex may be associated with DLBCL development and drug resistance. Among the four subunits of the GINS complex, GINS1 is the most important^20^; therefore, we focused our research on GINS1.

### GINS1 is a direct target of FOXP1 in DLBCL cell lines

**Figure 2:**
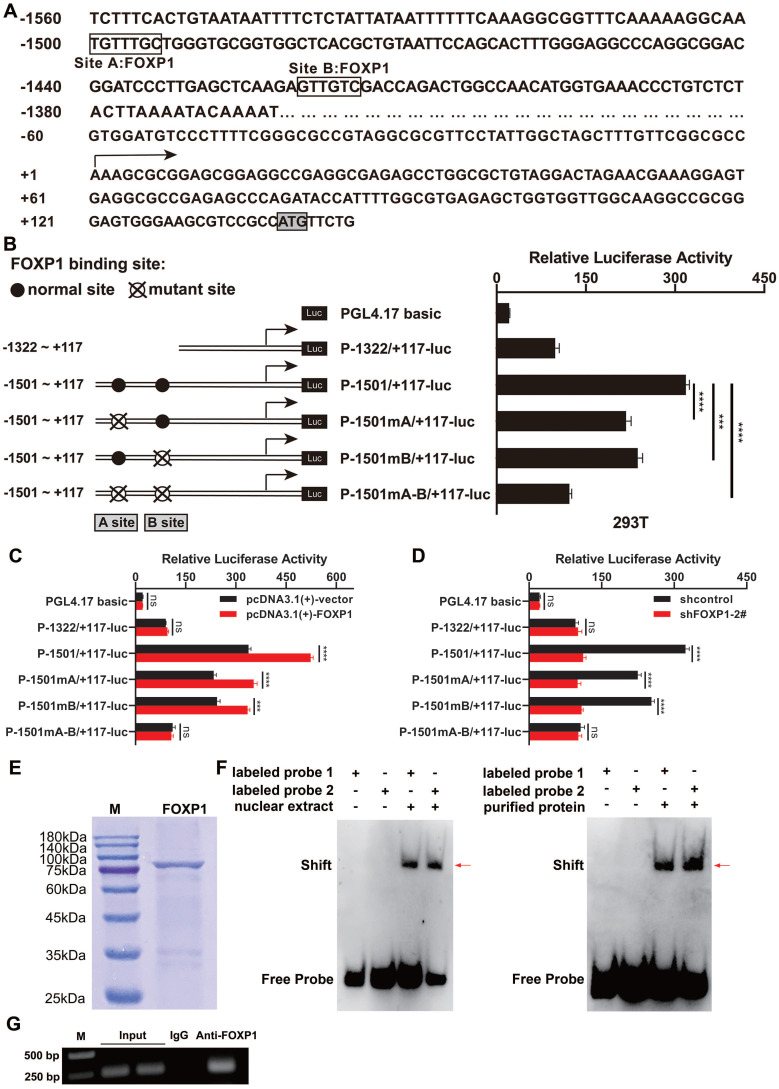
FOXP1 responsible for GINS1 expression in DLBCL. **(A)** Nucleotide sequence of the promoter region of GINS1 gene. Two predicted FOXP1 binding sites (site A and site B) were shown. +1 indicates the position of the transcription initiation site of the GINS1 gene. **(B)** Left: the schematic diagram of the luciferase reporter constructs containing the indicated genomic fragments of GINS1 gene was shown. Right: the results of the luciferase reporter assay in SU-DHL-2 cells. **C.** Co-transfected luciferase reporter constructs with pcDNA3.1-FOXP1. **D.** Co-transfected luciferase reporter constructs with shFOXP1 expressing plasmids. **E**. The recombinant FOXP1 were expressed and purified, then analyzed by SDS-PAGE. **F.** EMSA analysis with the SU-DHL-2 nuclear extract or recombinant FOXP1 protein.** (G)** ChIP assay. Agarose gel electrophoresis of ChIP products (***P<0.001).

First, to explore the mechanisms underlying the upregulation of GINS1 in DLBCL, promoter region was analyzed, and two FOXP1-binding sites were predicted by PROMO (Figure 2A). Then, these binding sites were analyzed by measuring the luciferase activity of the promoter reporter constructs (Figure 2B). The results showed that mutation at FOXP1 binding site A or binding site B (P-1501mA/+117-luc and P-1501mB/+117-luc) decreased GINS1 promoter activity in SU-DHL-2 cells. This finding indicates that two predicted sites, A and B, both contributed to GINS1 transcriptional activity. Consistent with these results, the promoter activity of P-1501/+117-luc was markedly activated by FOXP1 overexpression. However, when FOXP1-binding sites A and B were lost, overexpression of FOXP1 failed to stimulate promoter activity of P-1322/+117-luc (Figure 2C). Moreover, knockdown of FOXP1 significantly suppressed the transcriptional activity of GINS1 (Figure 2D).

Next, to further confirm the mechanism of FOXP1-mediated GINS1 transcription, recombinant FOXP1 protein was prepared for EMSA use (Figure 2E). EMSA results demonstrated that DNA‒protein complexes were detected when nuclear extracts of SU-DHL-2 cells or recombinant purified FOXP1 protein were incubated with probes containing FOXP1-binding site A or site B (Figure 2F). Moreover, ChIP assays also confirmed that FOXP1 could directly bind to the GINS1 promoter in SU-DHL-2 cells (Figure 2G). All these data suggested that FOXP1 was responsible for the transcription of GINS1 in DLBCL cells.

### The expression of GINS1 is positively correlated with FOXP1 expression in human DLBCL patient biopsies

**Figure 3:**
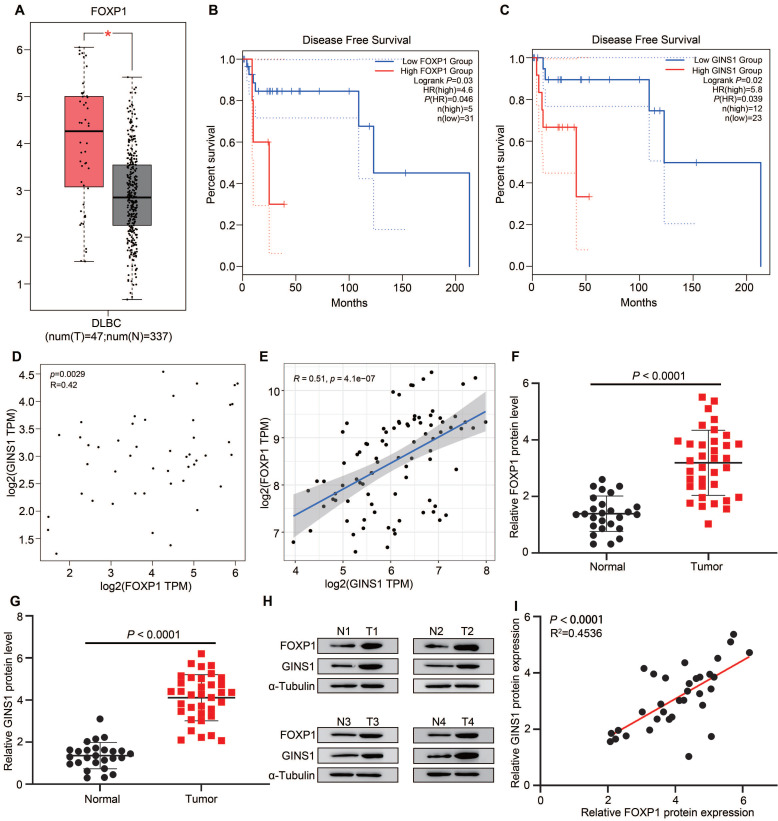
FOXP1 and GINS1 were both upregulated and correlated in DLBCL specimen. **A-D.** GEPIA analysis. A. FOXP1 expression in DLBCL tumor. **B-C.** Statistical analysis of FOXP1 or GINS1 for survival of DLBCL patients. D. The expression correlation of FOXP1 and GINS1 in DLBCL. **E.** The expression correlation of FOXP1 and GINS1 in GEO datasets GSE93984. F-I. Western blotting analysis of FOXP1 and GINS1 protein expression in our collected DLBCL tissues (T: DLBCL specimens (n=26), N: lymphadenitis patients (n=35). **F-G.** Western blot analysis.** H.** Representative western blotting analysis of GINS1 and FOXP1 protein expression. **I.** Scatter plots show a positive correlation between FOXP1 and GINS1 protein level in DLBCL samples.

To further explore the correlation of FOXP1 and GINS1 in DLBCL specimens, tissue expression and survival status were analyzed. First, we analyzed FOXP1 and GINS1 expression in data from The Cancer Genome Atlas (TCGA) by the GEPIA platform and found that both FOXP1 and GINS1 transcript levels were significantly higher in DLBCL samples than in normal samples (Figure 3A& Fig.1A). Kaplan-Meier survival analysis revealed that patients with high levels of FOXP1 or GINS1 exhibited shorter disease-free survival than patients with low levels of FOXP1 or GINS1 (Figure 3B-3C). The scatter plots showed that FOXP1 was positively correlated with GINS1 mRNA expression levels in DLBCL samples (Figure 3D). Moreover, the GSE93984 dataset also showed a positive correlation between FOXP1 and GINS1 expression. Next, we collected DLBCL samples at Nanjing First Hospital. High expression of GINS1 and FOXP1 was also detected in DLBCL patients (Fig.3 F-H). A positive correlation was obtained between the protein levels of FOXP1 and GINS1 (Figure 3I). Taken together, the current results showed that FOXP1 and GINS1 expression levels were significantly upregulated in DLBCL tissues, and FOXP1 was positively correlated with GINS1 expression. FOXP1 or GINS1 expression was strongly associated with inferior survival in DLBCL patients.

### The FOXP1/GINS1 regulatory axis was validated in DLBCL cell lines

**Figure 4:**
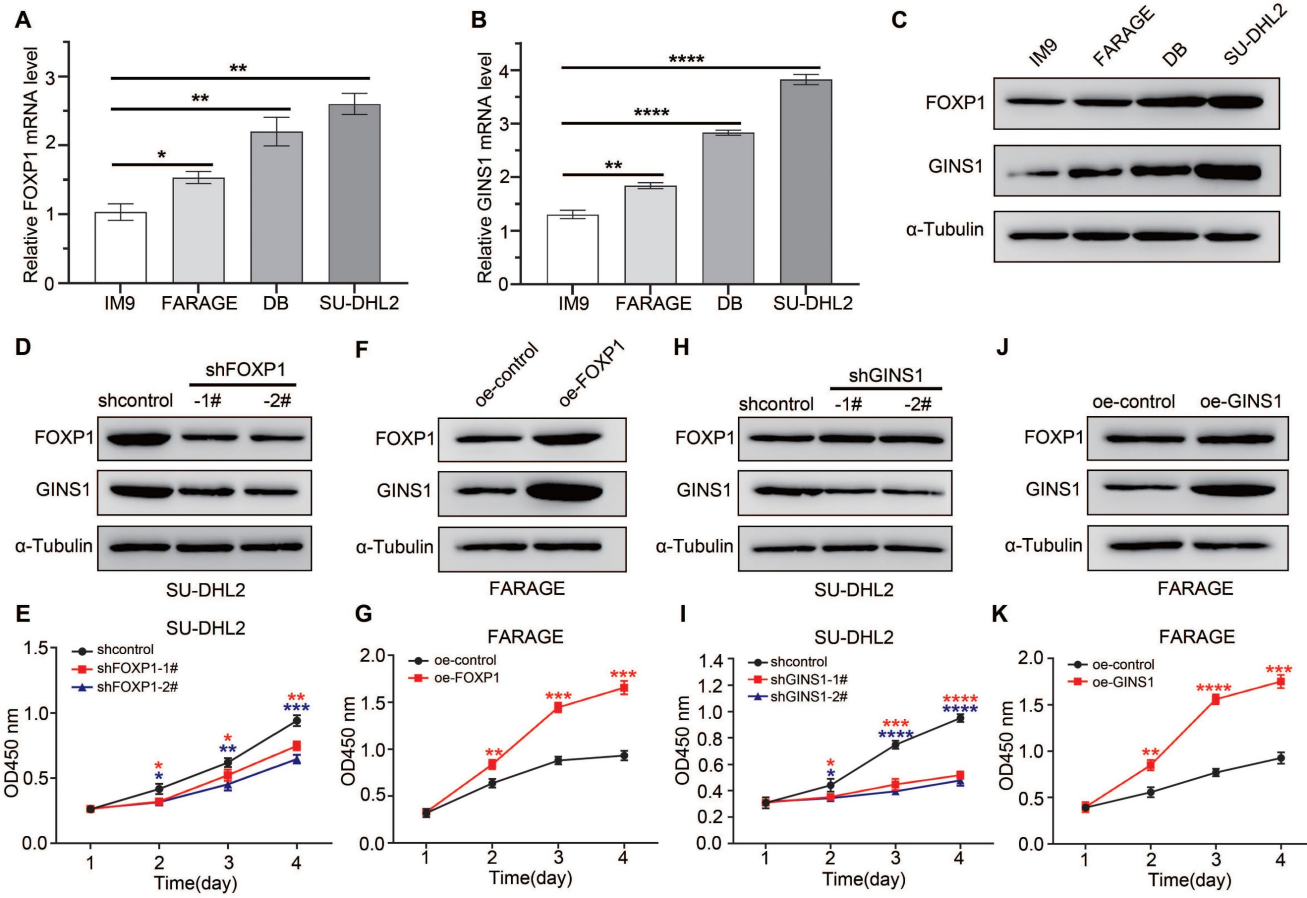
In DLBCL cell lines, FOXP1 expression level influenced GINS1 level, and affected cell growth. **A-C.** Analysis of GINS1 and FOXP1 expression in DLBCL cell lines. **A-B.** RT-qPCR analysis. **C.** western analysis. **D.** SU-DHL2 cells were infected with control (shcontrol) lentivirus or a lentivirus expressing shRNA for FOXP1. FOXP1 or GINS1 expression level were examined by western. **E.** CCK8 assay showing knockdown of FOXP1 inhibited proliferation in SU-DHL2 cell. **F.** FARAGE cells were infected with control lentivirus or lentivirus expressing FOXP1. FOXP1 or GINS1 expression level were examined by western. **G.** CCK8 assay. **H.** SU-DHL2 cells were infected with control (shcontrol) lentivirus or a lentivirus expressing shRNA for GINS1. GINS1 expression level were examined by western. **I.** CCK8 assay. **J.** FARAGE cells were infected with control lentivirus or a lentivirus expressing GINS1. GINS1 expression level were examined by western. **K.** Growth curves based on the CCK-8 results.

To further investigate whether FOXP1 regulates GINS1 expression in DLBCL cell lines, the expression levels of FOXP1 and GINS1 in the normal lymphocyte cell line IM9 and DLBCL cells (FARAGE, DB, and SU-DHL2) were measured using qRT‒PCR and western blotting. As shown in Figure 4A-4C, the expression levels of FOXP1 and GINS1 were obviously increased in DLBCL cells. Among the three tested DLBCL cell lines, SU-DHL2 cells presented the highest levels of both FOXP1 and GINS1, while FARAGE cells showed the lowest levels. Next, to unveil the effects of FOXP1 and GINS1 on DLBCL cells, SU-DHL2 cells were infected with lentiviral particles for FOXP1 gene silencing.

FARAGE cells were infected with lentiviral particles for FOXP1 overexpression. As shown in Figure 4D-4G, when FOXP1 was knocked down in SU-DHL2 cells, the GINS1 expression level was also decreased, and suppression of FOXP1 expression resulted in a significant reduction in the proliferation of SU-DHL2 cells. In contrast, while FOXP1 was overexpressed in FRAGE cells, the GINS1 expression level increased, and overexpression of FOXP1 observably promoted the proliferation of FRAGE cells as demonstrated by CCK8 assay. The direct influence of GINS1 on cell growth was investigated. SU-DHL2 cells were infected with lentiviral particles expressing GINS1-targeting shRNA, and FARAGE cells were infected with lentiviral particles overexpressing GINS1 (Fig.4H-4K). Western blot results confirmed that GINS1 expression was depressed in SU-DHL2 cells and increased in FARAGE cells after lentivirus infection. Compared with the control group, cell proliferation was significantly suppressed by downregulation of GINS1 in SU-DHL2 cells by CCK8 analysis. Meanwhile, overexpression of GINS1 observably promoted the proliferation of FRAGE cells. These results indicated the tumor-promoting function of GINS1 in DLBCL, which was consistent with our recently published reports^21^. Taken together, our data revealed that FOXP1 transcriptionally activates GINS1 expression by directly binding to the promoter region of the GINS1 gene. The FOXP1/GINS1 regulatory axis is important for DLBCL cell proliferation.

### The FOXP1/GINS1 regulatory axis was validated in a mouse model

**Figure 5:**
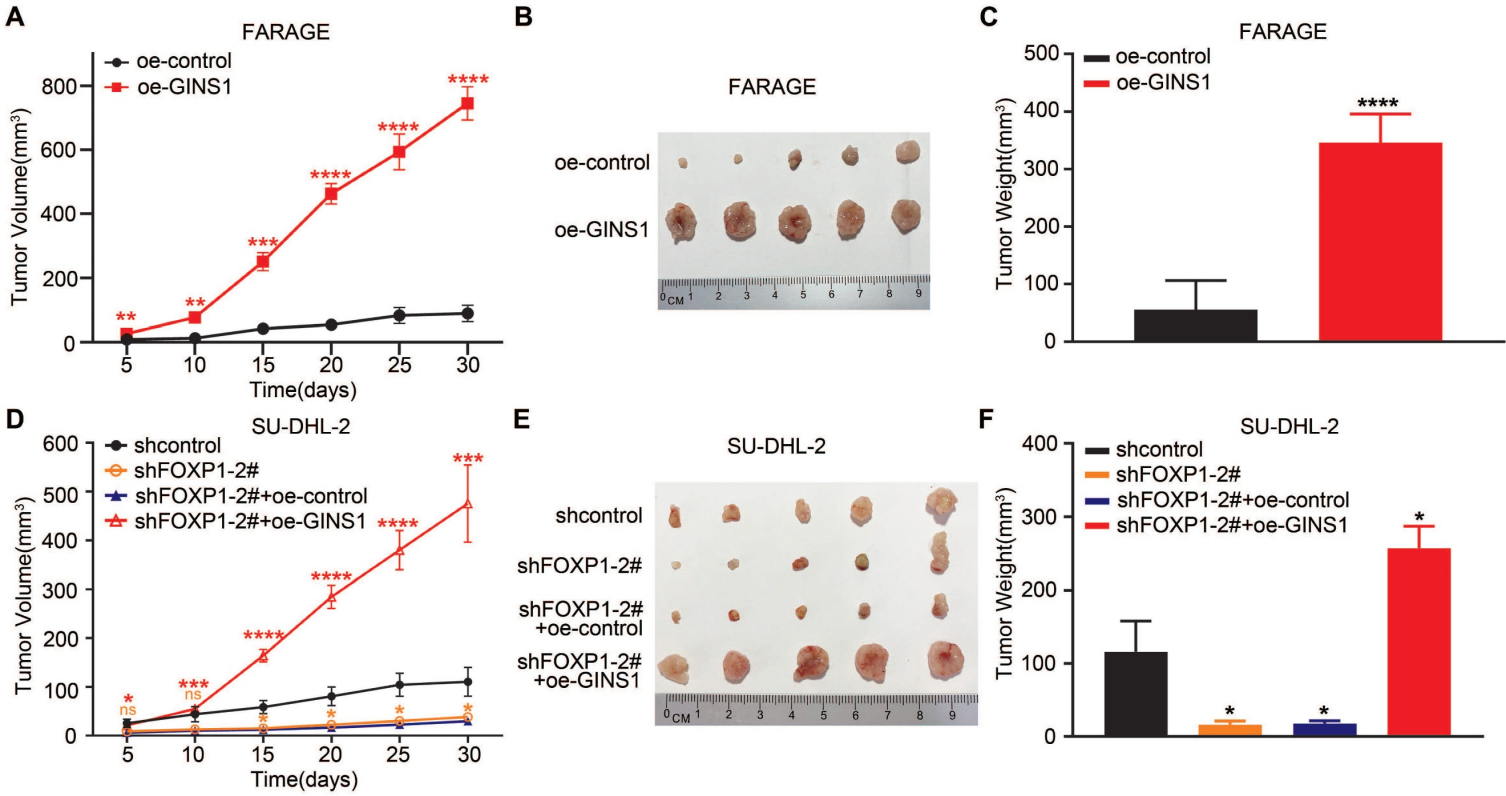
In vivo xenograft tumor model. **A-C.** Overexpression of GINS1 in Farage cells promoted cell proliferation in vivo. **A.** Tumor growth curve: subcutaneously injecting Farage-control cells, Farage-oe-GINS1 cells into the flank of nude mice, the tumor sizes of two groups were measured (five mice in each group). **B.** Tumor pictures. **C.** tumor weight. **D-F.** Knockdown of FOXP1 inhibited the proliferation of SU-DHL-2 cells. Over expression of GINS1 reversed the effect of FOXP1 silencing. **D.** Tumor growth curve. **E.** Tumor pictures. **F.** Tumor weight on day 30. (*P<0.05, **P<0.01, ***P<0.001).

To further validate the FOXP1-GINS1 axis in vivo, an in vivo xenograft lymphoma mouse model was established. FARAGE cells that overexpressed GINS1 were inoculated into nude mice. The results showed that GINS1 overexpression evidently increased tumor growth and tumor weight compared to the control group (Figure 5A-5C). Next, SU-DHL2-shFOXP1 cells with FOXP1 knockdown and control cells (SU-DHL2-shcontrol) were prepared. SU-DHL2-shFOXP1+oeGINS1 cells were also prepared to simultaneously silence FOXP1 and overexpress GINS1 in SU-DHL2 cells. The tumors formed by SU-DHL2-shFOXP1-1 cells showed decreased tumor size and weight compared to the control group (Figure 5D-5F). However, upregulation of GINS1 rescued the tumor volumes and weights suppressed by FOXP1- silencing in the SU-DHL2-shFOXP1+oeGINS1 groups, indicating that GINS1 overexpression reversed the growth inhibition induced by FOXP1 knockdown. These data suggested that the FOXP1/GINS1 regulatory axis was important for DLBCL tumor growth.

### Expression of GINS1 is associated with doxorubicin resistance

**Figure 6:**
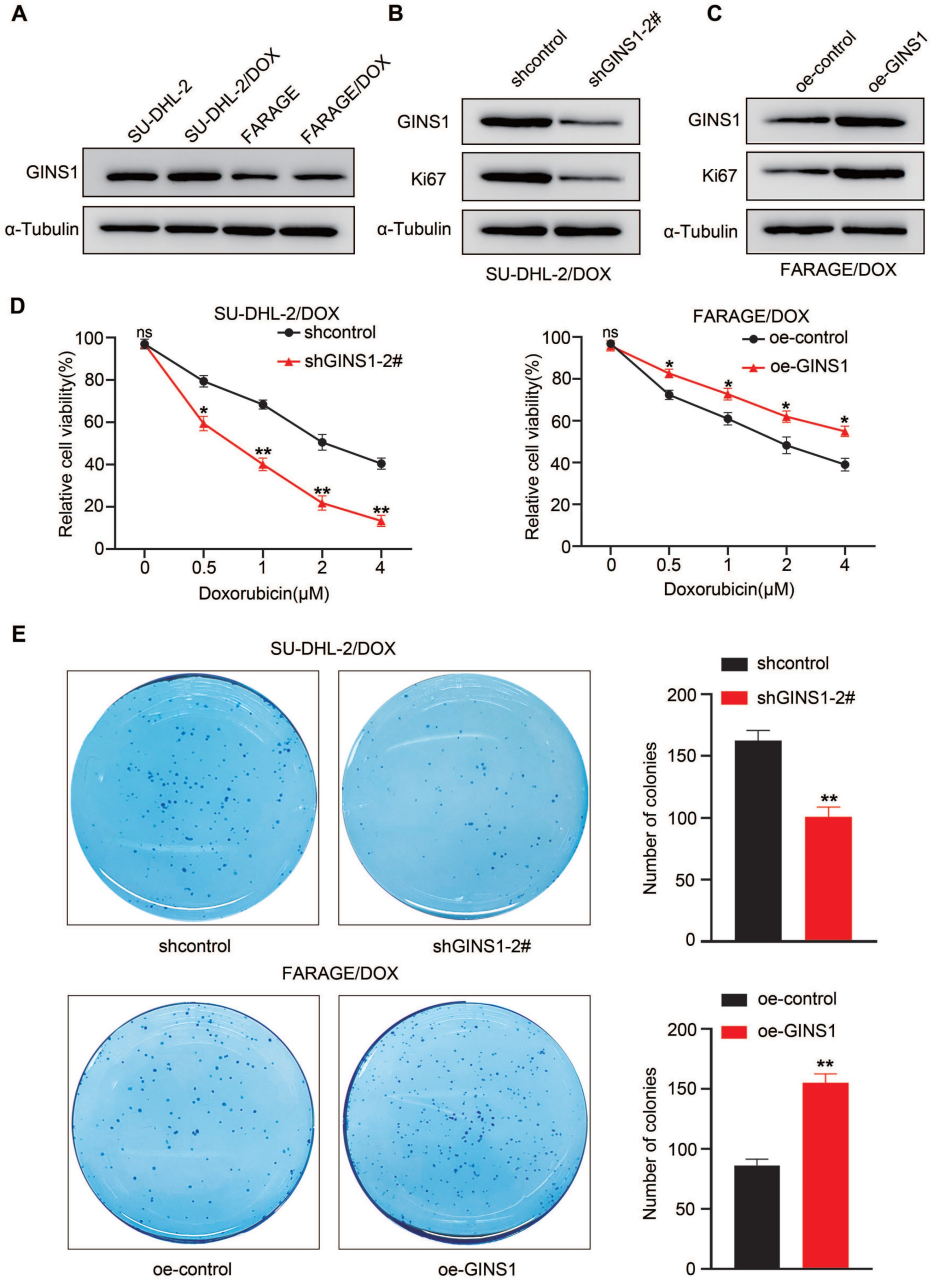
Silencing of GINS1 weakens DOX resistance in lymphoma cells. **A.** GINS1 protein in parental and DOX-resistant FARAGE and SU-DHL-2 cells examined by western blot analysis. **B-C.** FARAG/DOX and SU-DHL-2/DOX cells were infected with sh-GINS1 or oe-GINS1 expressing lentiviral particle, protein levels of GINS1 and Ki67 in FARAG/DOX and SU-DHL-2/DOX cells after lentiviral particle infection were examined by western blot analysis. **D.** Cell viability of FARAG/DOX and SU-DHL-2/DOX under different doses of DOX treatment was evaluated by CCK-8. **E.** Colony formation ability of FARAG/DOX and SU-DHL-2/DOX cells under 0.5μM DOX treatment examined by colony formation assay. Data represent mean values ± SEM of three independent experiments. *, P < 0.05.

The above results showed that elevated GINS1 expression was detected in lymphoma cells compared to normal lymphocytes. Next, to investigate whether GINS1 might affect the sensitivity of DLBCL cells to doxorubicin, an important component in the R-CHOP regimen, two DOX-resistant cell lines, FARAGE/DOX and SU-DHL-2/DOX, were induced. Doxorubicin (DOX) is a first-line chemotherapy medicine for DLBCL treatment. Doxorubicin resistance remains a major obstacle for treatment failure. Then, GINS1 expression was examined in the two DOX-resistant cell lines. Elevated GINS1 protein levels were detected compared to the parental cell lines (Fig.6A). These findings indicated that high GINS1 cells are less sensitive to doxorubicin, and GINS1 was potentially linked to DOX resistance in lymphoma. Therefore, knockdown and overexpression of GINS1 were performed through lentiviral particles. Western blot analysis showed that shGINS1 suppressed GINS1 and Ki67 protein levels in SU-DHL-2/DOX cells whereas oe-GINS1 elevated GINS1 and Ki67 protein levels in FARAGE/DOX cells (Fig. 6B-6C). Thereafter, CCK-8 assays were performed to examine the viability of cells after treatment with different doses of DOX to evaluate drug sensitivity. Silencing GINS1 caused a dramatic reduction in the survival of cells after doxorubicin treatment for 3 days, whereas GINS1 overexpression reduced the DOX sensitivity of FARAGE/DOX cells (Fig. 6D). Then, cells were treated with 0.5 μM DOX, and the colony formation assay showed that shGINS1 decreased, whereas oe-GINS1 increased, the number of colonies compared with the control lentiviral vector- infected FARAGE/DOX or SU-DHL-2/DOX cells (Fig. 6E). Taken together, our results showed that in DOX‑resistant cells, GINS1 shows a higher expression profile. Expression of GINS1 enhances the resistance of DLBCL cells to doxorubicin, and silencing of GINS1 weakens DOX resistance in lymphoma cells. Our data indicated that GINS1 is an important molecule that induces doxorubicin resistance.

### GINS1 upregulation restores DOX resistance in lymphoma cells suppressed by silencing FOXP1

**Figure 7:**
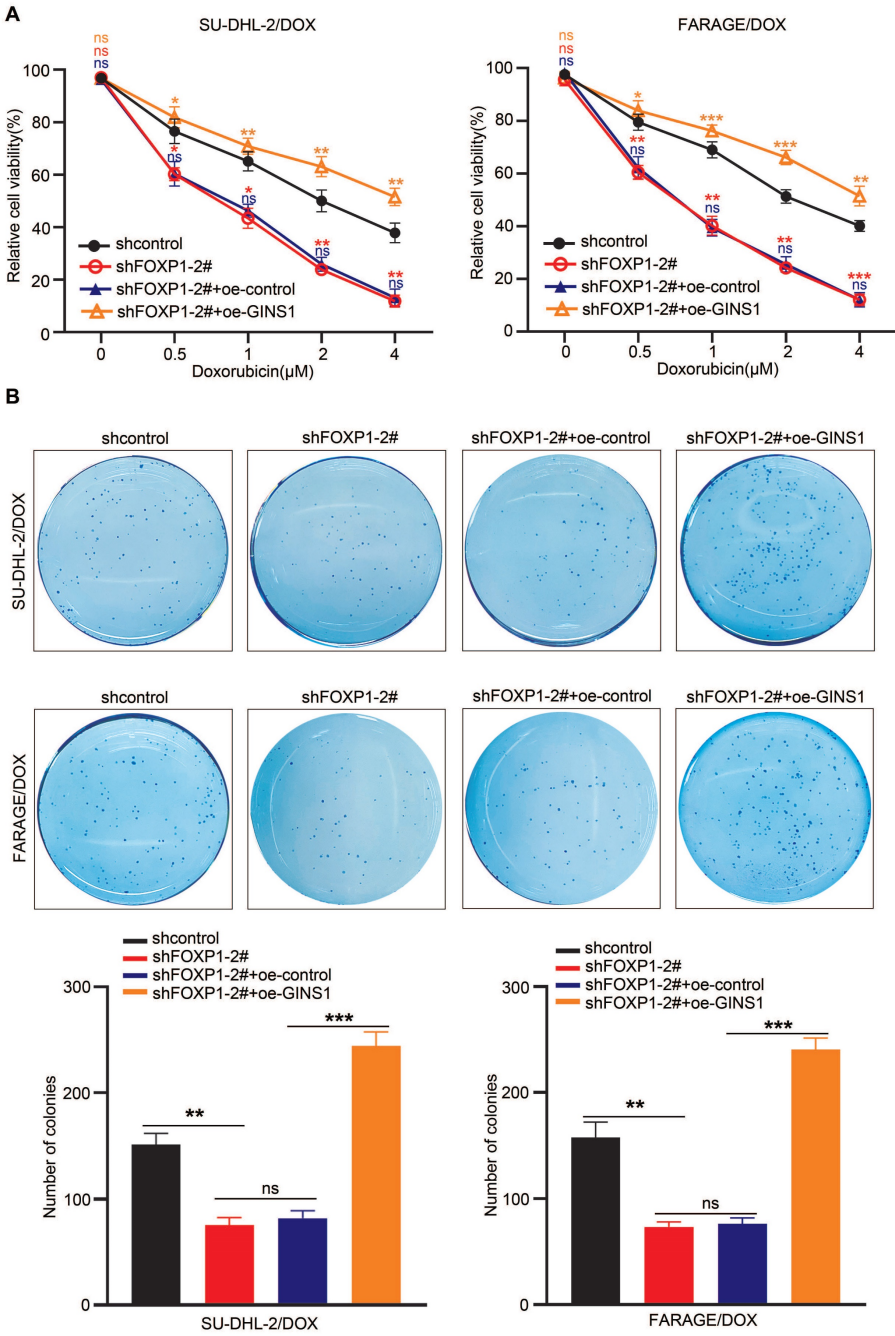
GINS1 upregulation restores DOX resistance in lymphoma cells suppressed by FOXP1 silencing. **A.** Cell viability of FARAG/DOX and SU-DHL-2/DOX cells under indicated doses of DOX treatment were analyzed by the CCK-8 method.** B.** Colony formation ability of FARAG/DOX and SU-DHL-2/DOX cells under 0.5 μM DOX treatment.

In view of the above finding that GINS1 enhances the survival of doxorubicin-challenged cells and the observation that GINS1 was regulated by FOXP1 and that both FOXP1 and GINS1 were positively correlated with poor prognosis of DLBCL, we next investigated whether upstream FOXP1 also contributed to DOX chemoresistance in DLBCL. Then, FOXP1 was silenced in FARAGE/DOX and SU-DHL-2/DOX cells, and the effects of doxorubicin on cell survival were examined. CCK-8 and colony formation results showed that FOXP1 silencing significantly increased the DOX sensitivity of these cells (Fig. 7A) and suppressed the colony formation ability of these two DOX resistant cell lines (Fig. 7B). Meanwhile, when GINS1 was overexpressed by lentivirus infection in these cells, GINS1 upregulation restored DOX resistance in lymphoma cells suppressed by FOXP1 silencing. These results indicated that the FOXP1/GINS1 axis is associated with DOX resistance in DLBCL.

## Discussion

Resistance to DOX is a serious challenge in the treatment of DLBCL, the chemoresistance mechanism of DLBCL is still poorly understood, and patient prognosis remains unsatisfactory. In this work, we demonstrated that FOXP1 mediated GINS1 upregulation and studied the effects of GINS1 on drug resistance in DLBCL for the first time. GINS1 is a crucial molecule that mediates doxorubicin resistance. Our research suggests the promising therapeutic potential of GINS1 in the clinical treatment of DLBCL.

The correlation between GINS1 and drug resistance has been studied in other malignancies. For instance, GINS1 was reported to function in hepatocarcinogenesis and sorafenib resistance^22^. High expression of GINS1 also promotes AraC resistance and cell cycle transit in leukemia cells^7^. In our previous research, it was revealed that aberrantly high GINS1 levels promoted cancer cell proliferation and indicated a poorer prognosis for patients with DLBCL^21^. In the current study, it was further revealed that overexpression of GINS1 conferred DOX-resistance to DLBCL cells.

Doxorubicin is the main DNA damage-inducing agent in conventional CHOP chemotherapy in patients with DLBCL. Previous studies have established that doxorubicin induces apoptosis of DLBCL cells as well as other tumor cells 23-26. The cytotoxicity of doxorubicin is due to a variety of mechanisms such as topoisomerase-II inhibition, DNA crosslinks and double-strand breaks^24^. More interestingly, it was recently reported that doxorubicin sensitizes glioblastoma cells in a GINS1-dependent manner^27^. GINS1 was also reported to have an anti-apoptotic function^28^. GINS1 is a member of the GINS complex. The GINS complex regulates both the initiation and progression of DNA replication. Therefore, GINS1 is pivotal for cell- cycle progression and cell proliferation. High expression of GINS1 not only induces cell cycle progression but also facilitates cell survival. This may be the mechanism by which GINS1 confers chemotherapy resistance to DLBCL cells. GINS1 was highly expressed in several types of leukemias, and knockdown of GINS1 reduced the growth of AML and CML cells 7. Our results suggested that GINS1 might be a potential therapeutic target to enhance the effect of chemotherapy in DLBCL.

In addition, FOXP1 was identified to activate GINS1 transcription by binding to its promoter and promoting GINS1 expression. Elevated expression of FOXP1 has been reported in DLBCL^29^-^32^, primary cutaneous large B-cell lymphomas (PCLBCLs)^33^, ^34^, follicular lymphoma^35^ and gastric mucosa-associated lymphoid tissue lymphoma (MALT)^36^. Consistently, FOXP1 depletion reduced the proliferation of hepatocellular carcinoma via G1/S phase arrest 37. Previous studies demonstrated that high FOXP1 protein expression can be the result of the chromosomal translocation t(3;14) or extra copies of FOXP1^32^, ^38^. The relationship between high FOXP1 protein expression and inferior outcome was identified previously. However, the underlying mechanism was not illustrated clearly. Here, we found that transcriptional activation of GINS1 by FOXP1 is involved in DLBCL proliferation and DOX-resistance. More interestingly, similar findings were also observed previously. FOXP1 was more highly expressed in clinical samples of patients predicted to be resistant to doxorubicin 39. High levels of miR-34a were associated with a better response to doxorubicin. The protective role of miR-34a is due to targeting of FOXP1. Our results also supported that downregulation of FOXP1 and its downstream GINS1 target sensitize DLBCL cells to doxorubicin. These findings reflected a mechanism of escape of DLBCL cells from chemotherapy and suggested that FOXP1-GINS1 may be a possible therapeutic target to enhance the effect of chemotherapy.

In summary, this study first demonstrated that FOXP1 and its target gene GINS1 contribute to DOX resistance in lymphoma cells. Our results confirm and extend previous reports linking FOXP1 overexpression to poor prognosis in DLBCL and may provide a potential FOXP1-GINS1 axis-based therapeutic strategy for reducing drug resistance in patients with DLBCL.

## Supplementary Material

Supplementary tables.
