# Supplementary material for: FOXP1-GINS1 axis promotes DLBCL proliferation and directs doxorubicin resistance
Source: J Cancer. 2023 Jul 16;14(12):2289–300. doi: 10.7150/jca.85906 (PMC10414051; doi:10.7150/jca.85906)
Supplement: Supplementary file 1 — Supplementary tables. [file jcav14p2289s1.pdf]

Supplementary table S1. Characteristics of the 35 patients with DLBCL

Characteristic	n	GINS1*		P value
		High expression (number of cases)	Low expression (number of cases)	
Total				
Gender				
Male	20	16	4	0.4505
Female	15	10	5	
Age				
<70	28	20	8	0.6478
≥ 70	7	6	1	
Stage				
I-II	9	3	6	**0.0033**
III-IV	26	23	3	
FOXP1*				
Positive	29	25	4	**0.0021**
Negative	6	1	5	

*FOXP1: The expression of FOXP1 was performed by Immunohistochemical staining. FOXP1 positive DLBCL was defined as 30% or more of the lymphoma cells showing distinct FOXP1 nuclear staining. For FOXP1-negative DLBCL cases, FOXP1 staining cells was less 30% of the lymphoma cells.

*GINS1: Western blotting was used to measure the GINS1 protein levels. ImageJ was used to compare the density of bands on western blot. Compared with the GINS1 average value of control people, when relative density values were above 2.5, then it was defined as high expression. While relative density values were below 2.5, then it was defined as low expression.

Supplementary table S2. Primers sequences and shRNA target sequences

Name	Sequences
Primer	
Human GINS1-Forward	5' CTTCAACGAGGATGGACT 3'
Human GINS1-Reverse	5' CATTTGGCAAGACGCTAC 3'
GAPDH Forward	5'TGAACGGGAAGCTCACTGG 3'
GAPDH Reverse	5'TCCACCACCCTGTTGCTGTA 3
FOXP1 Forward	5'TCAGTGGTAACCCTTCCCTTA 3'
FOXP1 Reverse	5'GTACAGGATGCACGGCTTG 3'
shRNA target sequences	
shcontrol	5' CCTAAGGTTAAGTCGCCCTCG 3'
shFOXP1-1	5' CAGCAGCAAGTTAGTGGATTA 3'
shFOXP1-2	5' CAGGCGGTACTCAGACAAATA 3'
shGINS1-1	5' TCAGGTGGACGAAGTGATT 3'
shGINS1-2	5' CCTGTATGACCGCTTGCTT 3'

Supplementary table S3. Plasmids

Reagent or Resource	Source	Identifier
pCDH-CMV-MCS-EF1-CopGFP-T2A-puro	Addgene	Cat#72263
pLP1	Miaolingbio	P0264
pLP2	Miaolingbio	P0265
pLP/VSVG	Miaolingbio	P0266
pLKO.1 puro	Addgene	Cat#8453
psPAX2	Addgene	Cat#12260
pMD2.G	Addgene	Cat#12259
pLKO.1-shGINS1 -1	This paper	N/A
pLKO.1-shGINS1 -2	This paper	N/A
PLKO.1-shFoxp1-1	This paper	N/A
PLKO.1-shFoxp1-2	This paper	N/A
pCDH- GINS1	This paper	N/A
pCDH- FOXP1	This paper	N/A
pcDNA 3.1(+) Foxp 1	This paper	N/A
pET28a-FOXP1	This paper	N/A
pGL-4.17 Basic	Promega	#9PIE672
P-1322/+117-LUC	This paper	N/A
P-1501/+117-LUC	This paper	N/A
P-1501mA/+117-LUC	This paper	N/A
P-1501mB/+117-LUC	This paper	N/A
P-1501mA-B/+117-LUC	This paper	N/A

Supplementary table S4. Promoter correlation probes and primers sequences

name	Sequences
Probe A sense	5’ TCAAAAAGGCAATGTTTGCTGGGTG 3’
Probe A antisense	5’ CACCCAGCAAACATTGCCTTTTTGA 3’
Probe B sense	5’ AGCTCAAGAGTTGTCGACCAGACTG 3’
Probe B antisense	5’ CAGTCTGGTCGACAACTCTTGAGCT 3’
ChIP-GINS1 sense	5’ GGCGGTTAGAAGCAAGATGG 3’
ChIP-GINS1 antisense	5’ ATTTTCCCCCCTCAGCCCTC 3’

Supplementary table S5. Chemicals and reagents

REAGENT or RESOURCE	SOURCE	IDENTIFIER
**Chemicals，consumables**		
TRIzol	Beyotime	Cat# R0016
BeyoRT™ II cDNA first chain synthesis kit	Beyotime	Cat# D7168L
IPTG	ThermoFisher	Cat# 34060
Firefly Luciferase Reporter Gene Assay Kit	Beyotime	Cat# RG005
β-galactosidase Assay Kit	Beyotime	Cat# RG0036
Chromatin Immunoprecipitation	Beyotime	Cat# P2078

(ChIP)Assay Kit		
Chemiluminescent EMSA Kit	Beyotime	Cat# GS009
Cell Counting Kit-8	Apexbio	Cat# K1018
SYBR® Premix Ex Taq™ II	Takara	Cat# DRR820A
Cell Culture Flask	NEST Biotechnology	Cat# 707003
50mL Min Bioreactor	NEST Biotechnology	Cat# 788211
**Antibodies**		
Anti-GINS1	Abcam	ab181112
Anti-a-Tubulin	Proteintech company	66031-1-Ig
Anti-Flag	Proteintech company	66008-4-Ig
Anti-FOXP1	Abcam	ab134055
Anti-Ki67	ThermoFisher	MA5-14520
Doxorubicin	Sigma-Aldrich	D1515
